# MiR-320-3p Regulates the Proliferation and Differentiation of Myogenic Progenitor Cells by Modulating Actin Remodeling

**DOI:** 10.3390/ijms23020801

**Published:** 2022-01-12

**Authors:** Mai Thi Nguyen, Wan Lee

**Affiliations:** 1 Department of Biochemistry, College of Medicine, Dongguk University, 123 Dongdae-ro, Gyeongju 38066, Korea; nguyenmainhp@gmail.com; 2 Channelopathy Research Center, College of Medicine, Dongguk University, 32 Dongguk-ro, Ilsan Dong-gu, Goyang 10326, Korea

**Keywords:** miR-320-3p, CFL2, YAP1, mechanotransduction, proliferation, differentiation

## Abstract

Skeletal myogenesis is essential for the maintenance of muscle quality and quantity, and impaired myogenesis is intimately associated with muscle wasting diseases. Although microRNA (miRNA) plays a crucial role in myogenesis and relates to muscle wasting in obesity, the molecular targets and roles of miRNAs modulated by saturated fatty acids (SFA) are largely unknown. In the present study, we investigated the role of miR-320-3p on the differentiation of myogenic progenitor cells. Palmitic acid (PA), the most abundant dietary SFA, suppressed myogenic factors expression and impaired differentiation in C2C12 myoblasts, and these effects were accompanied by CFL2 downregulation and miR-320-3p upregulation. In particular, miR-320-3p appeared to target *CFL2* mRNA directly and suppress the expression of CFL2, an essential factor for filamentous actin (F-actin) depolymerization. Transfection of myoblasts with miR-320-3p mimic increased F-actin formation and nuclear translocation of Yes-associated protein 1 (YAP1), a key component of mechanotransduction. Furthermore, miR-320-3p mimic increased myoblast proliferation and markedly impeded the expression of MyoD and MyoG, consequently inhibiting myoblast differentiation. In conclusion, our current study highlights the role of miR-320-3p on CFL2 expression, YAP1 activation, and myoblast differentiation and suggests that PA-inducible miR-320-3p is a significant mediator of muscle wasting in obesity.

## 1. Introduction

Skeletal myogenesis is a tightly regulated complex process required for maintaining muscle mass and integrity, which are essential for the appropriate physical and metabolic function of the body [1]. Accordingly, impairment of skeletal myogenesis provokes muscle wasting diseases that increase the risk of morbidity and mortality, especially in older people [2]. Muscle wasting is closely associated with various conditions that suppress myogenesis, including oxidative stress, apoptosis, and senescence [3]. In addition, numerous studies have indicated that obesity and high saturated fatty acids (SFA) intake enhance fat accumulation in muscle and eventually lead to lipotoxicity and muscle wasting [4,5]. Furthermore, accumulating evidence suggests that microRNAs (miRNAs) modulated by SFA and obesity contribute to the pathogenesis of muscle wasting [6,7]. However, the molecular mechanism responsible for the regulation of myogenesis by SFA-induced miRNAs remains to be elucidated.

MiRNAs are a class of endogenous short non-coding RNAs that function as negative regulators of gene expression [8]. During the past decade, a growing body of research has unveiled the diverse functions of miRNAs in skeletal muscle biology, such as myogenesis, maintenance, regeneration, and myopathies [9,10]. Interestingly, numerous miRNAs have been shown to be dysregulated in obesity and involved in the pathogenesis of muscle wasting via the inhibition of myogenesis [6,11]. Among those, miR-320a (miR-320-3p) has been suggested to be a potential mediator for lipid and glucose metabolism-associated diseases and adiposity [12]. MiR-320-3p is a member of the miR-320 family (miR-320a/-b/-c/-d/-e) and is the most widely distributed in all primates [13]. Since the miR-320 family plays a multifaceted role in cell proliferation, apoptosis, and metastasis, previous studies have mainly focused on the significance of miR-320-3p on oncogenesis and cancer progression [14]. Nevertheless, in addition to the relation to obesity and metabolic diseases [12], miR-320-3p has been linked to several conditions resulting in muscle wasting, such as oxidative stress, ER stress, mitochondrial dysfunction, apoptosis, and enhanced autophagy [14,15,16,17,18]. Hence, these findings imply that miR-320-3p may be implicated in myogenesis and muscle homeostasis by regulating cell proliferation, differentiation, and regeneration. However, the roles and significance of miR-320-3p in myogenic differentiation have not been explored yet. 

Actin dynamics modulated by actin-binding proteins control cytoskeleton remodeling required for myogenesis [19,20]. Cofilins are actin-modulating proteins that belong to the actin depolymerizing factor (ADF)/cofilin family and are required for actin remodeling in response to various signals [21]. In particular, a skeletal muscle-specific cofilin 2 (CFL2) is essential for the formation and maintenance of skeletal muscle through actin cytoskeleton remodeling by depolymerizing filamentous actin (F-actin) [22]. CFL2 knockout mice were fatal within seven days of birth because of skeletal muscle weakening and insufficiency [23] and exhibited sarcomere disruption due to loss of actin depolymerization [24]. Furthermore, it has been proposed that CFL-mediated actin remodeling regulates cell proliferation, which is associated with myogenic differentiation [25,26]. Interestingly, we previously showed that CFL2 knockdown impeded the differentiation of C2C12 myoblasts by enhancing cell proliferation and cell cycle progression [27]. However, although CFL2 is an essential component of actin remodeling and skeletal myogenesis, little is known about the role of miR-320-3p in CFL2 expression, actin remodeling, and myogenic differentiation.

Here, the roles played by an SFA-inducible miRNA were investigated on the regulation of CFL2 and the differentiation of C2C12 myoblasts. We revealed that palmitic acid (PA)-inducible miR-320-3p suppressed CFL2 expression, increased F-actin, and activated Yes-associated protein 1 (YAP1) in myoblasts. Furthermore, we also demonstrated the significance of miR-320-3p in myoblast proliferation, myogenic regulatory factors expression, and differentiation. These findings highlight the crucial regulatory roles of miR-320-3p in myogenesis via CFL2/F-actin/YAP1 mechanotransduction axis and provide a possible mechanism for the miRNA-mediated myogenic regulation in obesity.

## 2. Results

### 2.1. PA Impaired Differentiation but Induced miR-320-3p in Myoblasts

**Figure 1:**
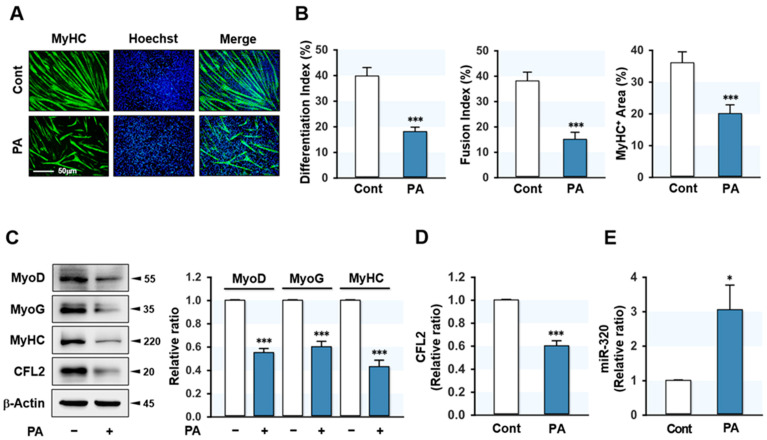
Effect of PA on myoblast differentiation and miR-320-3p expression. C2C12 cells were treated with BSA (Cont) or BSA-conjugated PA (PA, 100 μM) for 24 h and induced differentiation. (**A**) Immunocytochemical staining of MyHC (green) at day 5 of differentiation. Nuclei were stained with Hoechst 33342 (blue). Scale bar, 50 μm. (**B**) MyHC-positive area, differentiation index, and fusion index were quantitated with ImageJ software. (**C**,**D**) The expressions of myogenic regulatory factors (MyoG and MyoD), MyHC, and CFL2 were analyzed by immunoblotting at day 3 of differentiation. The values between panels indicate relative protein levels normalized against β-Actin. (**E**) The mRNA levels of miR-320-3p were measured by *q*RT-PCR 24 h after transfection and normalized against U6. Results are means ± SEMs (*n* > 3). *, *p* < 0.05; ***, *p* < 0.001 vs. control.

We previously reported that CFL2 is required for myogenic differentiation of progenitor cells [27]. Therefore, in this study, we investigated whether SFA affects CFL2 expression and differentiation in myoblasts. C2C12 cells were pretreated with 100 μM of PA for 24 h, and the myogenic factors expressions and myoblast differentiation were assessed at day 3 and day 5 of differentiation, respectively. According to immunocytochemistry (Figure 1A,B), PA dramatically impeded the differentiation, fusion, and myotube formation of C2C12 myoblasts. Moreover, PA markedly reduced myogenic factors in myoblasts such as MyoD, MyoG, and MyHC, with concomitant suppression of CFL2 (Figure 1C,D) required for myogenesis and actin dynamics. Accordingly, it was postulated that specific miRNAs upregulated by PA might play a role in the downregulation of CFL2. Accumulating evidence suggests that miR-320-3p is upregulated in obesity and functions as a potential mediator for lipid and glucose metabolism-associated diseases and adiposity [12]. Moreover, miR-320-3p is found to target 3′UTR of *CFL2* based on the miRNA target prediction analysis by TargetScan and Pictar. Hence, we next confirmed the induction of miR-320-3p expression in the PA-treated cells by *q*RT-PCR (Figure 1E), and miR-320-3p was selected for further investigations in the regulation of CFL2 expression and myoblast differentiation. 

### 2.2. CFL2 Is a Direct Target of miR-320-3p

**Figure 2:**
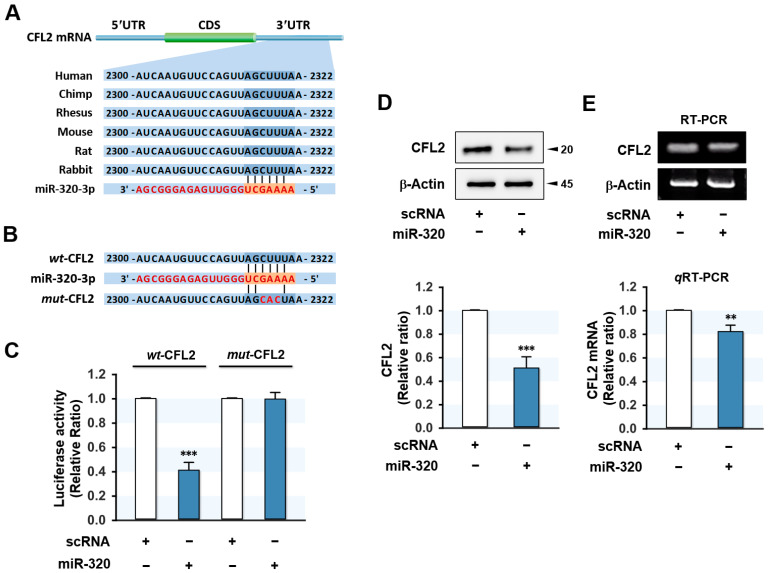
MiR-320-3p downregulated CFL2 expression. (**A**) The conservation of miR-320-3p targeting site in the *CFL2* 3′UTR. (**B**) The potential binding site for the miR-320-3p seed sequence in the wild-type (*wt*-CFL2) or mutant (*mut*-CFL2) 3′UTR fragment of *CFL2*. (**C**) The relative luciferase activity in myoblasts cotransfected with either *wt*-CFL2 or *mut*-CFL2 plasmids and scRNA or miR-320-3p mimic was analyzed 24 h after transfection. (**D**) CFL2 protein expression was determined in myoblasts 24 h after transfection with scRNA or miR-320-3p mimic. The values between panels indicate relative protein levels normalized against β-Actin. (**E**) The mRNA level of *CFL2* was analyzed by RT-PCR (upper panel) and *q*RT-PCR (lower panel), and the values normalized against U6. Results are means ± SEMs (*n* > 3). **, *p* < 0.01; ***, *p* < 0.001 vs. scRNA control.

Since the miR-320-3p expression was inversely associated with CFL2 expression in myoblasts, we investigated whether miR-320-3p regulates CFL2 directly. The 3′UTR of *CFL2* has a potential binding site for miR-320-3p, as shown in Figure 2A. To determine CFL2 as a direct target gene of miR-320-3p, we cloned segments of *CFL2* 3′UTR containing either a wild-type (*wt*-CFL2) or mutant (*mut*-CFL2) binding site for miR-320-3p into pmirGLO luciferase reporter plasmid (Figure 2B) and then cotransfected miR-320-3p mimic or scRNA control with pmirGLO constructs into C2C12 cells. As expected, miR-320-3p mimic significantly inhibited luciferase activity in the *wt*-CFL2, but not in the *mut*-CFL2 (Figure 2C). Thus, luciferase reporter analysis confirmed the direct binding of miR-320-3p to the 3′UTR of *CFL2*. Next, we investigated whether miR-320-3p transfection suppresses CFL2 in C2C12 myoblasts. As shown in Figure 2D, miR-320-3p mimic reduced the protein expression of CFL2 markedly as compared with scRNA. Furthermore, miR-320-3p mimic also decreased *CFL2* mRNA expression (Figure 2E), which indicated miR-320-3p negatively regulates CFL2 expression.

### 2.3. MiR-320-3p Increased F-Actin and Nuclear YAP1

**Figure 3:**
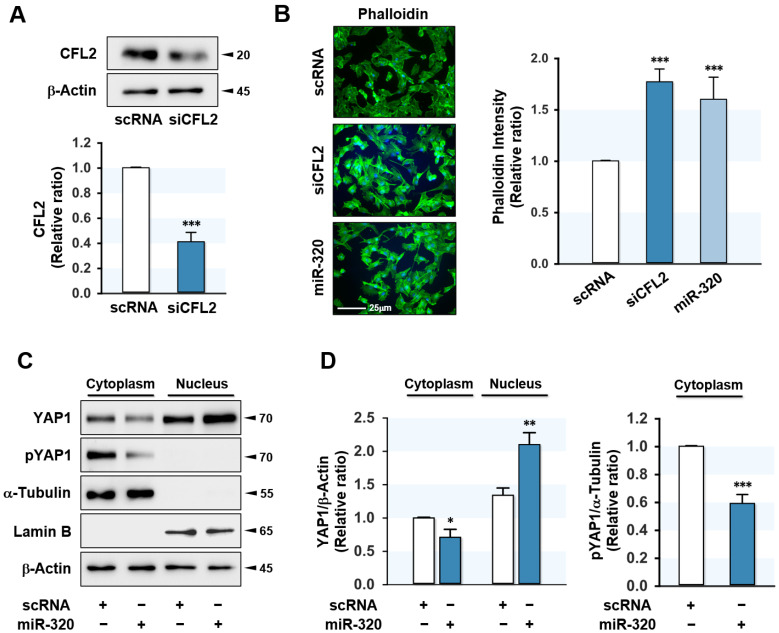
MiR-320-3p enhanced F-actin and nuclear YAP1. C2C12 cells were transfected with scRNA, CFL2 siRNA (siCFL2), or miR-320-3p mimic (miR-320) for 24 h. (**A**) The silencing efficiency of siCFL2 on CFL2 protein was determined by immunoblotting. The values between panels indicate relative protein levels normalized against β-Actin. (**B**) F-actin was stained with FITC-phalloidin (green), and nuclei were stained with Hoechst 33342 (blue). Scale bar, 25 μm. Relative intensities of phalloidin were quantitated with ImageJ software. (**C**,**D**) The expression of nuclear and cytoplasmic YAP1 and phosphorylated YAP1 (pYAP1) was determined by immunoblotting. Lamin B and α-Tubulin were used for nuclear and cytoplasmic markers, respectively. The values between panels indicate relative protein levels normalized against Lamin B or α-Tubulin. Results are means ± SEMs (*n* > 3). *, *p* < 0.05; **, *p* < 0.01; ***, *p* < 0.001 vs. scRNA control.

Previously, it was reported that the knockdown of CFL2 enhanced F-actin accumulation in myoblasts [27] because CFL2 is required for actin depolymerization [26]. Accordingly, we hypothesized that induction of miR-320-3p expression would lead to F-actin accumulation by suppressing CFL2. Under our experimental condition, transfection of myoblasts with miR-320-3p mimic and siCFL2 effectively lowered CFL2 protein expressions by 50% (Figure 2D) and 60% (Figure 3A), respectively. As expected, miR-320-3p mimic and siCFL2 dramatically augmented F-actin in myoblasts (Figure 3B). Given that the amount of total actin remained consistent regardless of transfected oligonucleotides, the accumulation of F-actin by miR-320-3p mimic appeared to be the consequence of a defect in actin depolymerization resulting from CFL2 reduction. It has been known that F-actin stimulates the nuclear translocation of a transcriptional coactivator YAP1 by suppressing its phosphorylation, thereby modulating mechanotransduction in the Hippo signaling pathway and enhancing cell proliferation through the activation of proliferative transcriptional programs [28]. In this study, miR-320-3p mimic significantly inhibited the phosphorylation of YAP1 (pYAP1) in the cytoplasm. Subsequently, it increased the translocation of YAP1 from the cytoplasm to the nucleus (Figure 3C,D), indicating miR-320-3p can induce the F-actin-mediated nuclear translocation of YAP1 in the Hippo signaling pathway.

### 2.4. MiR-320-3p Activated the Proliferation of Myoblasts

**Figure 4:**
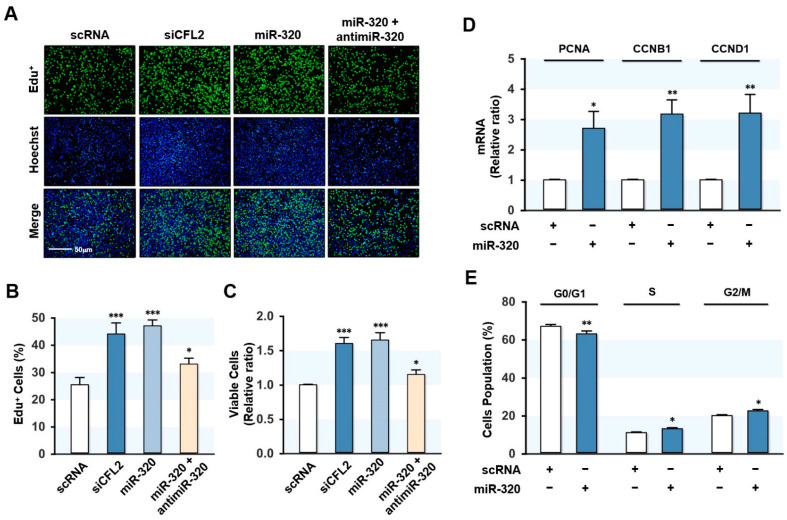
MiR-320-3p promoted cell proliferation and cell cycle progression. C2C12 cells were transfected with scRNA, CFL2 siRNA (siCFL2), miR-320-3p mimic (miR-320), or antimiR-320-3p (antimiR-320) for 24 h. (**A**) Representative images of EdU incorporation analysis. Cells during DNA replication were labeled with EdU (green), and nuclei were stained with Hoechst 33342 (blue). Scale bar, 50 µm. (**B**) The percentage of EdU-positive cells was quantitated with ImageJ software. (**C**) Viable cells were stained and analyzed using a cell viability assay kit. (**D**) The mRNA level of YAP1 target genes, such as CCNB1, CCND1, and PCNA, was analyzed by *q*RT-PCR, and the values normalized against U6. (**E**) Flow cytometry analysis at 24 h after transfection. Results are means ± SEMs (*n* > 3). *, *p* < 0.05; **, *p* < 0.01; ***, *p* < 0.001 vs. scRNA control.

We next investigated the role of miR-320-3p in myoblast proliferation and cell cycle progression because miR-320-3p activated YAP1 in the Hippo signaling pathway. We determined myoblast proliferation by evaluating EdU incorporation 24 h after siCFL2 or miR-320-3p mimic transfection. Knockdown of CFL2 by siCFL2 caused a robust increase in EdU-positive cells (Figure 4A,B), consistent with our recent report [27]. Interestingly, transfection with miR-320-3p mimic also significantly enhanced the proportion of EdU-positive cells, while antimiR-320-3p cotransfection almost entirely abrogated the enhanced EdU incorporation exerted by miR-320-3p mimic (Figure 4A,B). Viable cells count also confirmed that miR-320-3p has a stimulatory effect on myoblast proliferation (Figure 4C). We then assessed the expressions of YAP1 target genes associated with cell cycle and proliferation by *q*RT-PCR analysis and found that miR-320-3p mimic significantly increased PCNA, CCNB1, and CCND1 mRNA levels (Figure 4D). Therefore, we next analyzed the cell cycle by a FACS. Transfection with miR-320-3p mimic reduced the percentage of cells in the G0/G1 phase but enhanced the proportion of cells in the S and G2/M phases (Figure 4E). Overall, miR-320-3p mimic led to the transcriptional activations of YAP1 target genes and enhanced cell proliferation and cell cycle progression.

### 2.5. MiR-320-3p Suppressed Myogenic Factors Expression

**Figure 5:**
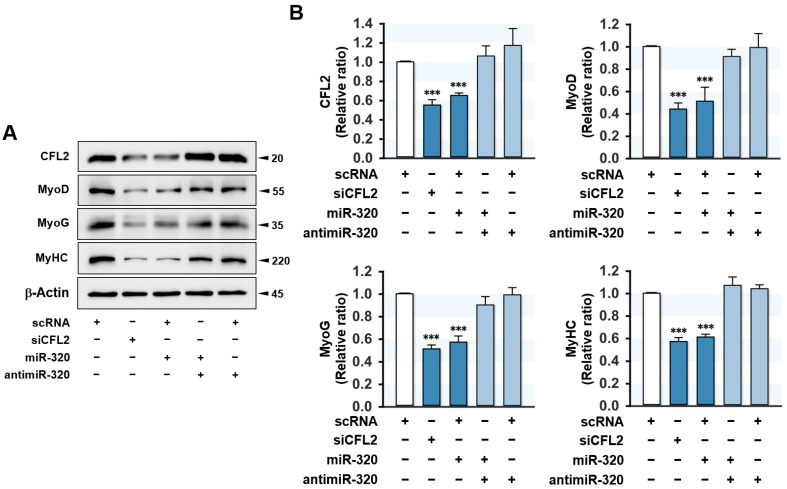
MiR-320-3p inhibited myogenic factors expressions. Cells were transfected with scRNA, siCFL2, miR-320-3p mimic (miR-320), or antimiR-320-3p (antimiR-320) and differentiated for 3 days. (**A**) Immunoblotting analysis of myogenic factors, such as MyHC, MyoD, MyoG, and CFL2. (**B**) The values between panels indicate relative protein levels normalized against β-Actin. Results are means ± SEMs (*n* > 3). ***, *p* < 0.001 vs. scRNA control.

Since the exit of the cell cycle of proliferating myoblasts is recognized as prerequisite conditions for the differentiation of myogenic progenitor cells [1], the promotion of proliferation and cell cycle in myoblasts by miR-320-3p may inhibit myogenic differentiation. Above all, to examine the effect of miR-320-3p on myogenic factors expression, the cellular levels of MyoD, MyoG, and MyHC were measured at day 3 of differentiation (Figure 5). Transfection with siCFL2 decreased CFL2 expression by approximately 50% and dramatically reduced MyoD, MyoG, and MyHC protein levels compared to scRNA control. Similarly, miR-320-3p mimic also drastically inhibited CFL2 expression by 40% and reduced myogenic factors expression. Furthermore, cotransfection with miR-320-3p and antimiR-320 abolished the suppressive effect of miR-320-3p on myogenic factors expression, suggesting that ectopic expression of miR-320-3p resulted in the downregulation of myogenic factors in myoblasts.

### 2.6. MiR-320-3p Impeded Myoblast Differentiation

**Figure 6:**
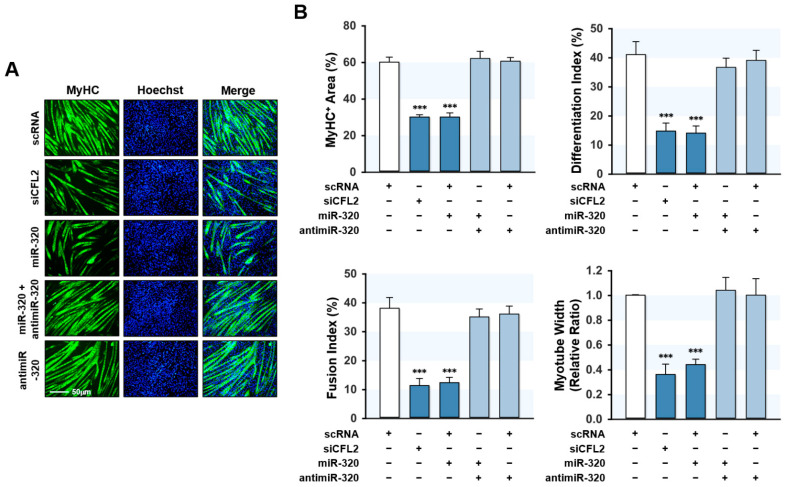
MiR-320-3p impeded the differentiation of myoblasts. Cells were transfected with scRNA, siCFL2, miR-320-3p mimic (miR-320), or antimiR-320-3p (antimiR-320) and differentiated for 5 days. (**A**) Immunocytochemical staining of MyHC (green) at day 5 of differentiation. Nuclei were stained with Hoechst 33342 (blue). Scale bar, 50 μm. (**B**) MyHC-positive area, myotube width, differentiation index, and fusion index were quantitated with ImageJ software. Results are means ± SEMs (*n* > 3). ***, *p* < 0.001 vs. scRNA control.

The effects of the miR-320-3p on myogenic factors expression imply that miR-320-3p may function as a negative regulator of myoblast differentiation and myotube formation. Therefore, we next examined the differentiation of myoblasts at day 5 of differentiation. Myogenic differentiation and myotube formation were visualized by immunocytochemistry and determined quantitatively using ImageJ software (Figure 6A,B). Based on MyHC immunofluorescence, transfection with siCFL2 robustly hindered myotube formation in myoblasts. According to quantitative analysis, including the percentage area of MyHC-positive cells, differentiation index, fusion index, and myotube width, indicated that CFL2 knockdown significantly inhibited myoblast differentiation. Likewise, miR-320-3p mimic also drastically impeded the myogenic differentiation of C2C12 cells. Moreover, cotransfection with miR-320-3p and antimiR-320-3p almost entirely abolished the inhibitory effect of miR-320-3p mimic on myogenic differentiation in myoblasts. Collectively, our findings suggest that miR-320-3p plays a negative regulatory role in myoblast differentiation and myotube formation. 

## 3. Discussion

Despite the recent progress in knowledge of miRNAs on muscle biology, the molecular targets and roles of miRNAs modulated by SFA or obesity are largely unknown in myogenesis. Previously, we have shown that CFL2 knockdown hindered the differentiation of myoblasts through enhancing cell proliferation [27]. This study reveals a vital role of miR-320-3p in the regulations on CFL2 expression and myogenic differentiation, which supports our hypothesis that SFA-inducible miRNAs impair myogenesis and contribute to the pathogenesis of muscle wasting. It should be noted that miR-320-3p markedly stimulated myoblast proliferation and cell cycle progression, which led to impaired myogenic differentiation (Figure 3 and Figure 4). Since the proliferation and differentiation of myogenic progenitor cells are inversely modulated during myogenesis, exiting the cell cycle and proliferation of myoblasts are prerequisites for differentiation and myotube formation [1,29]. In this aspect, the effect of miR-320-3p on the cell cycle and proliferation is causally related to the inhibition of myoblast differentiation. Previous studies have shown the dysregulation of miR-320-3p in various malignancies and its diverse role on cell proliferation and cell cycle. MiR-320-3p frequently appears to function as a tumor suppressor by inhibiting proliferation, invasion, and migration [14]. In contrast, several studies have demonstrated that miR-320-3p increases proliferation and promotes cell cycle progression in various cells. For example, miR-320-3p regulated tumorigenesis by increasing proliferation, invasion, metastasis of pancreatic cancer cells [30]. In addition, transfection of miR-320 promoted embryonic stem cell proliferation by suppressing p57 and p21 and facilitating cell cycle progression [31]. These findings are consistent with previous studies in which miR-320-3p overexpression promoted the proliferation of cerebral neurons [32], B cells [33], and ovarian cancer cells [34]. More recently, Costa et al. found that stable expression of miR-320a increased cell proliferation, whereas stable silencing of miR-320a decreased cell proliferation in malignant mesothelioma cells [35]. Nevertheless, the discrepancy between tumor-suppressive or proliferative functions of miR-320-3p in various cancers might be ascribed to the differences in the composition and abundance of protein components and the expression level miR-320-3p in the diverse cell types. From this point of view, it is worth noting that CFL2 is skeletal muscle-specific and gradually upregulated during myogenic progenitor cell differentiation. Moreover, miR-320-3p has been reported to be increased in various conditions associated with muscle wasting [12], and the Hippo signaling pathway is linked to the regulation of skeletal muscle growth and wasting in many ways [36]. Therefore, the activation of YAP1 in mechanotransduction triggered by miR-320-3p-mediated CFL2 suppression seems to be a distinct feature in myogenic progenitor cells and skeletal muscle as compared to other cell types.

Then how does miR-320-3p enhance myoblast proliferation? It should be highlighted that miR-320-3p increased F-actin formation by directly targeting CFL2. CFL2 has been recognized as an essential component of actin remodeling and mechanical stress modulation in the cytoskeleton [26,37]. Actin dynamics are involved in the activation of YAP1 in the Hippo signaling pathway that regulates organ sizes by coordinating progenitor proliferation and differentiation [28,38]. In the Hippo pathway, the dephosphorylation of cytosolic YAP1 induces nuclear translocation, which triggers proliferative transcriptional processes as a mechanotransduction mechanism [39]. Previously, F-actin was found to reduce the phosphorylation of YAP1, increase nuclear YAP1, and stimulate cell proliferation consequently [28,40]. Moreover, CFL2 functions as a negative regulator of YAP1 by increasing its phosphorylations in the cytosol [25,41]. As a result, CFL-mediated actin remodeling is crucial for the YAP1 mechanotransduction and cell proliferation [25,26]. Our previous study demonstrated that suppression of CFL2 increased F-actin, cell cycle progression, and proliferation in myoblasts [27]. Similarly, CFL2 depletion also was found to increase F-actin formation and nuclear translocation of YAP1 in cardiomyocytes [42]. Furthermore, cytochalasin D, a powerful actin depolymerizer, impeded the activation of YAP1, while jasplakinolide, an actin polymerizer, increased nuclear YAP1 [42]. Our current study indicates that cell proliferation enhanced by miR-320-3p is mainly related to YAP1 activation via mechanotransduction resulting from CFL2 reduction. Thus, miR-320-3p increases F-actin accumulation by suppressing CFL2, activates YAP1, provokes myoblast proliferation, and thereby impairs myoblast differentiation. 

Although the mechanism by which PA increases miR-320-3p expression is currently unknown and warrants further investigation, certain transcription factors activated in obesity could cause miR-320-3p transcriptional activation in myoblasts. Based on in silico analysis of transcription factor binding sequences, the promoter of miR-320-3p has putative binding sites for several transcription factors, such as YY1, ELK1, and p53, which are associated with obesity. Indeed, it has been shown that these transcription factors are involved in the induction of miR-320-3p in various cells. A previous study exhibited that transcription factors YY1 and ELK1 bound to the miR-320-3p promoter and increased miR-320-3p expression in cervical cancer and colon cancer cells [43]. A recent study also reported the role of p53 in miR-320-3p upregulation in mesothelioma cells [35]. Interestingly, these transcription factors have been known to be activated by obesity, adiposity, or a high-fat diet in various studies. The expression of YY1, a ubiquitous multifunctional transcription factor, was markedly increased in the PA-treated hepatocytes and mice fatty liver induced by streptozotocin and HFD [44]. Furthermore, YY1 was upregulated in the livers of HFD-induced obese mice and NAFLD patients [45]. In addition, ELK1, a member of the ternary complex factor subfamily of ETS-domain transcription factors, was increased in visceral adipose tissues of obese human subjects [46]. Furthermore, the activation of p53, which is known to induce insulin resistance through multiple tissues/organs, was intimately associated with lipid accumulation and obesity in humans and mice [47]. Although further study is required to determine the transcriptional activation of miR-320-3p expression by the transcription factors described above, YY1, ELK1, and p53 transcription factors in a background of obesity may be involved in the induction of miR-320-3p.

## 4. Materials and Methods

### 4.1. Cell Culture and PA Treatment

C2C12 myoblasts (ATCC, Manassas, VA, USA), a mouse myogenic progenitor cell line, were grown in a growth medium (GM; 10% fetal bovine serum (FBS)-containing Dulbecco’s modified Eagle’s medium (DMEM) supplemented with antibiotics (penicillin-streptomycin, 1%, Gibco, Carlsbad, CA, USA) at 37 °C in a 5% CO_2_ humidified condition. The myoblasts were then cultured on 35 mm plates at a density of 1.3 × 10^5^ in 2 mL of GM. When confluency reached 80 to 90%, a differentiation medium (DM) composed of horse serum (2%, dialyzed, Gibco) in DMEM supplemented with 1% penicillin-streptomycin was used to induce cell differentiation. The medium was refreshed every 24 h. When necessary, cells were pretreated with either bovine serum albumin (BSA) or BSA-conjugated PA (100 μM) in GM for 24 h before differentiation. All reagents and materials were obtained from Sigma-Aldrich unless otherwise specified.

### 4.2. Transfection of Oligonucleotides 

The oligonucleotides, such as siRNA of CFL2 (siCFL2), miR-320-3p mimic, miR-320-3p inhibitor (antimiR-320-3p; a 2′-O-methyl modified antisense oligonucleotide against miR-320-3p), and scrambled control RNA (scRNA), were purchased from Genolution (Seoul, Korea). The oligonucleotide at a final concentration of 200 nM were transiently transfected into cells using Lipofectamine 2000 (Invitrogen, Carlsbad, CA, USA) following the manufacturer’s instructions. All oligonucleotide sequences used in this study are listed in Table S1.

### 4.3. RNA Preparation and Quantitative Real-Time PCR (qRT-PCR) 

Total RNA was extracted 24 h after transfection using miRNeasy Mini Kit and Qiazol reagent (Qiagen) according to the manufacturer’s protocol. RNAs quality and quantity were verified by gel electrophoresis and a UV-1700 PharmaSpec spectrophotometer (Shimadzu, Japan). The iTaq polymerase (Promega) and SYBR Green I kit were used to determine the expressions of miRNAs and other target genes by *q*RT-PCR in LightCycler 480 (Roche Applied Science, Penzberg, Germany) using 2^−ΔΔCt^ method with normalization to U6. The sequences of all primers and reaction conditions used for RT-PCR and *q*RT-PCR are described in Table S2.

### 4.4. Dual-Luciferase Reporter Analysis

The fragment of *CFL2* 3′UTR (315 nt, *wt*-CFL2) containing a miR-320-3p binding site was subcloned into the pmirGLO vector (Promega) to produce a wild-type reporter. The site-directed mutation was conducted by amplifying the wild-type reporter plasmid using overlapping oligonucleotides that contained mutations in miR-320-3p binding sites (*mut*-CFL2). The primer sequences for subcloning and mutagenesis are shown in Table S2. For dual-luciferase reporter analysis, C2C12 cells were cotransfected with a pmirGLO plasmid containing either *wt*-CFL2 or *mut*-CFL2 and miR-320-3p or scRNA using Lipofectamine 2000. The luciferase activity was determined using a Dual-Luciferase Reporter Assay System (Abcam, Cambridge, UK) 24 h after transfection. 

### 4.5. Immunoblot Analysis 

Cells were lysed by phosphate-buffered saline (PBS) with Triton X-100 (2%), PMSF (1%), and 0.2 mM phosphatase inhibitor cocktail 2 (Sigma-Aldrich, St. Louis, MO, USA) on ice. If necessary, NE-PER Nuclear and Cytoplasmic Extraction Kit (Thermo Fisher Scientific, Waltham, MA, USA) was used to extract cytoplasmic and nuclear protein fractions. Protein concentration was measured by the Bradford method, and 20 μg of protein was resolved by SDS-PAGE, transferred to nitrocellulose membranes (Amersham Biosciences, Piscataway, NJ, USA), and blotted with specific antibodies (Table S3) after blocking with 5% skim milk (Becton, France). The protein bands were visualized with a Femto reagent (Thermo Fisher Scientific, Waltham, MA, USA) and analyzed using Fusion Solo Chemiluminescence Imaging System (Vilber, France) equipped with Evolution Capt software (Vilber, France).

### 4.6. Immunofluorescence Staining Analysis

After transfecting C2C12 cells and inducing differentiation for five days, the cells were fixed with paraformaldehyde (4%), permeabilized with Triton X-100 (0.3%), and blocked with BSA (3%) in PBS for 2 h. The processed cells were incubated overnight with a MyHC antibody at 4 °C. The next day, an anti-mouse secondary antibody conjugated with Alexa-488 (Invitrogen) was applied for 1.5 h. Nuclei were counterstained with Hoechst 33342 (Invitrogen). F-actin staining was achieved by incubating cells with FITC-conjugated phalloidin (P5282, Sigma) at 50 μg/mL [27]. Images were captured by a Leica fluorescence microscope. Differentiation index (percentage of nuclei in MyHC-positive myotubes/total nuclei) and fusion index (percentage of nuclei in MyHC-positive myotubes with ≥3 nuclei/total nuclei) were calculated as described previously [27]. The images were analyzed using ImageJ software.

### 4.7. Cell Proliferation Assays

For cell proliferation assay, the transfected cells were labeled with EdU (10 µM) from the Click-iT™ EdU assay kit (Invitrogen) for 4 h at 37 °C, then fixed, permeabilized, and treated with a Click-iT reaction cocktail. Hoechst 33342 was used for nuclei staining, and all images were captured by a Leica fluorescent microscope. The proportions of EdU-positive cells were calculated from at least five randomly chosen areas using ImageJ software, and at least three independent experiments were conducted. For viable cell counting, C2C12 cells were seeded on a 96-well plate and then transfected with indicated oligonucleotides. After 24 h, 10 μL Quanti-max^TM^ WST-8 Cell Viability Assay Kit (BioMax, Seoul, Korea) was added to each well and incubated at 37 °C for 4 h. Finally, the absorbance was analyzed at 450 nm using a microplate reader (Model 680, Bio-rad, Hercules, CA, USA).

### 4.8. Flow Cytometry

The transfected cells were washed with PBS and fixed with ethanol (70%) at 4 °C overnight. After removing the supernatants, the pellets were incubated with 500 µL Cell Cycle kit reagent (C03551, Beckman Coulter, Brea, CA, USA) at room temperature for 20 min in the dark. A CytoFLEX (Beckman Coulter) was used for cell cycle analysis.

### 4.9. Bioinformatic and Statistical Analysis

The potential target binding sites on the 3′UTR of *CFL2* mRNA for miR-320-3p were predicted using bioinformatics tools, such as TargetScan (www.targetscan.org accessed on 1 November 2021) and Pictar (pictar.mdc-berlin.de accessed on 1 November 2021). Statistical analysis was performed using a two-tailed unpaired Student’s *t*-test to compare the difference between two groups. Statistical analysis was performed using the Student’s *t*-test for unpaired data. Results are presented as the means ± standard errors obtained from at least three independent experiments.

## 5. Conclusions

This study demonstrated that miR-320-3p is an essential regulator in actin remodeling, mechanotransduction, and differentiation of myogenic progenitor cells. It was shown that PA impeded the differentiation of C2C12 myoblasts together with CFL2 reduction and miR-320-3p induction. Indeed, miR-320-3p directly targeted 3′UTR of *CFL2* and inhibited CFL2 expression. Furthermore, MiR-320-3p mimic augmented F-actin, induced YAP1 nuclear translocation, promoted myoblast proliferation, and eventually inhibited the expressions of myogenic factors and myoblast differentiation. These functions of miR-320-3p indicate the existence of a novel miRNA-mediated regulatory mechanism in myogenesis. Thus, miR-320-3p may be a critical mediator in the relationship between obesity and muscle wasting, allowing for the development of effective diagnostic and treatment methods for muscle wasting in obesity. 

## Data Availability

The data presented in this study are available on request from the corresponding author.

## Supplementary Materials

The following are available online at https://www.mdpi.com/article/10.3390/ijms23020801/s1.
