# Supplementary material for: MiR-320-3p Regulates the Proliferation and Differentiation of Myogenic Progenitor Cells by Modulating Actin Remodeling
Source: Int J Mol Sci. 2022 Jan 12;23(2):801. doi: 10.3390/ijms23020801 (PMC8775871; doi:10.3390/ijms23020801)
Supplement: Supplementary file 1 [file ijms-23-00801-s001.zip › ijms-1478157-supplementary.pdf]

Table S1. Oligonucleotide sequences for transfection

Gene	Primer sequence (5´-3´)
scRNA (control RNA)	UCACAACCUCCUAGAAAGAGUAGA
siCFL2	GCUCUAAAGAUGCCAUUAAUU
miR-320-3p	AAAAGCUGGGUUGAGAGGGCGA
antimiR-320	UCGCCCUCUCAACCCAGCUUUU

Table S2. Primer lists and conditions for *q*RT-PCR, RT-PCR and cloning

(A) Mouse primer lists for *q*RT-PCR and RT-PCR

Gene	Primer sequence (5′-3′)		Product size	Annealing Temperature	Concentration		Cycle
					cDNA	Primer	
miR-320-3p	F.P	AAAAGCTGGGTTGAGAGGGCGA	90	55	2 ng/μl	0.5 μM	40
miRNA universal Primer	R.P	miScript universal primer (Qiagen)					
U6	F.P	CTCGCTTCGGCAGCACA	94				
	R.P	AACGCTTCACGAATTTGCGT					
β-Actin	F.P	TCACCCACACTGTGCCCATCTACGA	348	58			
	R.P	GGATGCCACAGGATTCCATACCCA					
CFL2	F.P	CCGACCCCTCCTTCTTCTCG	100	58			
	R.P	GTAACTCCAGATGCCATAGTG					
CCNB1	F.P	GAGCTATCCTCATTGACTGG	125	58			
	R.P	CATCTTCTTGGGCACACAAC					
CCND1	F.P	ACCAATCTCCTCAACGACCG	228	58			
	R.P	ACGGAAGGGAAGAGAAGGG					
PCNA	F.P	GAACCTGCAGAGCATGGACTC	201	58			
	R.P	GGTGTCTGCATTATCTTCAGCCC					

(B) Primer lists for wild-type and mutant 3'UTR cloning

Gene	Primer sequence (5′-3′)		Product size	Annealing Temperature	Concentration		Cycle
					cDNA	Primer	
CFL2*wt*	F.P	CCTTAACTTTCTTGTATGCCTCTAG	315	58	2 ng/μl	0.5 μM	35
	R.P	AGCAAGCTAGCAGTAAAATATTGCC					
CFL2*mut*	F.P	CCAGTTAGCACCAAAAGG	72				
	R.P	AGCAAGCTAGCAGTAAAATATTGCC					
	F.P	CCTTAACTTTCTTGTATGCCTCTAG	262				
	R.P	ACCTTTTGGTGCTAACTGG					

Table S3. Antibodies list

Antibody	Type	Targeted species	Manufacturer	Cat. No.	Dilution ratio*
CFL2	Polyclonal	Rabbit	Lifespan Biosciences, Seatlle, WT, USA	LS-C409553	1:2,000
MyHC	Monoclonal	Mouse	DSHB, Iowa, IA, USA	MF20	1:1,000
MyoD	Monoclonal	Mouse	Santa Cruz Biotechnology, Dallas, TX, USA	sc-377460	1:1,000
MyoG	Monoclonal	Mouse	Santa Cruz Biotechnology, Dallas, TX, USA	sc-12732	1:1,000
YAP1	Monoclonal	Rabbit	Cell Signaling Technology, Danvers, MA, USA	14074S	1:10,000
pYAP1	Polyclonal	Rabbit	Cell Signaling Technology, Danvers, MA, USA	4911S	1:10,000
Lamin B	Monoclonal	Rabbit	Abcam, Cambridge, United Kingdom	ab151735	1:2,500
α-Tubulin	Monoclonal	Mouse	DSHB, Iowa, IA, USA	12G10	1:2,000
β-Actin	Monoclonal	Rabbit	Sigma-Aldrich Chemical, St. Louis USA	A2066	1:10,000
Antibodies HRP-linked anti-rabbit IgG			Cell Signaling Technology, Danvers, MA, USA	#7074	1:10,000
Goat anti-mouse(H+L)			Thermo Fisher Scientific, Waltham, MA, USA	#32430	1:2,000

*All blots were visualized using a Femto reagent (Thermo Fisher Scientific, Waltham, MA, USA).
